# Some food toxic for pets

**DOI:** 10.2478/v10102-009-0012-4

**Published:** 2009-09-28

**Authors:** Natália Kovalkovičová, Irena Šutiaková, Juraj Pistl, Václav Šutiak

**Affiliations:** 1 University of Veterinary Medicine, Komenského 73, 041 81 Košice, Slovak Republic; 2 University of Prešov, Ul. 17. Novembra 1, 081 16 Prešov, Slovak Republic

**Keywords:** pet, human food, intoxication

## Abstract

According to world statistics, dogs and cats are the species that owners most frequently seek assistance with potential poisonings, accounting 95–98% of all reported animal cases. Exposures occur more commonly in the summer and in December that is associated with the holiday season. The majority (>90%) of animal poisonings are accidental and acute in nature and occur near or at the animal owner's home. Feeding human foodstuff to pets may also prove dangerous for their health.

The aim of this review was to present common food items that should not be fed (intentionally or unintentionally) to dogs, i.e. chocolate, caffeine, and other methylxanthines, grapes, raisins, onion, garlic, avocado, alcohol, nuts, xylitol contained in chewing gum and candies, etc. Onion and avocado are toxic for cats, too. The clinical effects of individual toxicants and possible therapy are also mentioned. Knowing what human food has the potential to be involved in serious toxicoses should allow veterinarians to better educate their clients on means of preventing pet poisonings.

It can be concluded that the best advice must surely be to give animal fodder or treats specifically developed for their diets.

## Introduction

There is an unlimited number of agents by which exposed animals may become poisoned, and for the most part, which specific agents are involved in animal poisonings will be dependent upon what is available in the animals' environment, the potential or inclination for the animal to be exposed to the agent, the amount of agent to which the animal is exposed, and the individual sensitivity of the animal to the effects of the agent (Gupta, 2007).

Nowadys, the most common agents involved in animal exposures are rodenticides, chocolate, pharmaceuticals, glycols, metals, pesticides, plants, miscellaneous agents. Rodenticides, chocolate (approximately one quarter of all exposures) and pharmaceuticals (22% of exposures) make up the majority of agents in the most recent reports (Cope *et al*., 2006; Martinez-Haro *et al*., 2008). Feeding human foodstuff to pets may also prove dangerous for their health.

A difference between species regarding the occurrence of poisonings was found and such fact can be explained by different behaviour between dogs and cats.

Perhaps at least partly because of their inquisitive natures and willingness to investigate everything with their mouths, dogs far outrank other species when it comes to owners seeking aid for potential poisonings, making up 70–80% of all animal cases (Gupta, 2007). There are also some foods, which are edible for humans, and even other species of animals, that can pose hazards for dogs because of their different metabolism, e.g. chocolate, caffeine and other methylxanthines, grapes, raisins, onion, garlic, avocado, alcohol, nuts, etc.

Due, perhaps, to the more discriminating habits and appetites, cats account for only 11–20% of reported animal exposures to potential toxicants, which is three times less frequent than dogs. The cats are independent and less restrict to a definite space. Consequently, they are more susceptible to become victims of poisoning when tasteless and odorless toxic agents are mixed with tasty foods. In spite of being very selective in their alimentary pattern, cats can not notice the presence of the poison mixed with food, as insecticide aldicarb that is often mixed with fish that has strong odour and flavour (Xavier *et al*., 2007). Cats may due to their grooming habits, be more susceptible to toxicants that come into contact with their fur, this is especially problematic with agents to which cats are exquisitely sensitive (e.g. ethylene glycol) (Gupta, 2007). The most common poisonous foods for cats are onion and garlic and other related root vegetables, green tomatoes, green raw pottatoes, chocolate, grapes and raisins, etc.

Some food may cause only mild digestive upsets, whereas, others can cause severe illness, and even death in pets. Knowing what agents have the potential to be involved in serious toxicoses should allow veterinarians to better educate their clients on means of preventing animal poisonings through the appropriate use of household products and the removal of potential hazards from the animals' environments.

## Chocolate, caffeine and other methylxanthines

Chocolate is derived from the roasted seeds of *Theobroma cacao* and its toxic principles are the methylxanthines theobromine (3,7-dimethylxanthine) and caffeine (1,3,7-trimethylxanthine). Theobromine is also found in tea, cola beverages, and some other foods.

Chocolate toxicoses occur especially at holidays: Valentine's day, Easter, Halloween, and Christmas and may result in potentially life-threatening cardiac arrhythmias and CNS dysfunction (Stidworthy *et al*., 1997; Beasley, 1999).

### Sources

Most poisonings from methylxanthines occur as a result of chocolate ingestion. Chocolate is toxic to all species, especially to smaller dogs, though a toxic dose will vary depending on factors like whether the dog ate the chocolate on an empty stomach, if the dog is particularly sensitive to chocolate, and the type of chocolate, since dark chocolate is more toxic, whereas milk chocolate less so, and white chocolate must be consumed in extremely large quantities to cause a serious problem. Contributing factors include indiscriminate eating habits and readily available sources of chocolate. Deaths have also been reported in livestock fed cocoa byproducts and in animals consuming mulch from cocoa-bean hulls. Cocoa bean hulls or waste used as bedding for animals has caused toxicosis primarily in horses. Third most prevalent poisoning from these agents is a result of caffeine tablets ingested by dogs or hype race horses (tablets often contain 100 mg each). Theophylline tablets or elixirs are also used as human or veterinary medication.

### The methylxanthine content

The most important toxic component of chocolate – the methylxanthine alkaloid theobromine is present in variable concentrations dependent on the quality of the chocolate – the darker or richer in cocoa solids the more dangerous the preparation. Cocoa powder and cooking chocolate are the most toxic forms (Sutton, 1981). Table 1 shows the total methylxanthine concentration in chocolate (*www.merckvetmanual.com*).

**Table 1:** The total methylxanthine concentration in chocolate (www.merckvetmanual.com).

Type of product	The total methylxanthine concentration
dry cocoa powder	28.5 mg/g
unsweetened (baker's) chocolate	16 mg/g
cocoa bean hulls	9.1 mg/g; 0.5–0.85% theobromine
semisweet chocolate and sweet dark chocolate	5.4–5.7 mg/g
milk chocolate	2.3 mg/g
refined chocolate candies	1.4–2.1 g/kg
white chocolate	an insignificant source of methylxanthines
cocoa beans	1–2% theobromine

The amount of theobromine found in chocolate is small enough that chocolate can be safely consumed by humans in large quantities, but animals that metabolize theobromine more slowly can easily consume enough chocolate to cause poisoning. Although the concentration of theobromine in chocolate is 3–10 times that of caffeine, both constituents contribute to the clinical syndrome seen in chocolate toxicosis.

### Toxic doses

A 10-kilogram dog can be seriously affected if it eats a quarter of a 250 g packet of cocoa powder or half of a 250 g block of cooking chocolate. These forms of chocolate contain ten times more theobromine than milk chocolate. Thus, a chocolate mud cake could be a real health risk for a small dog. Even licking a substantial part of the chocolate icing from a cake can make a dog unwell. A dog needs to eat more than a 250 g block of milk chocolate to be affected. Obviously, the smaller the dog, the less it needs to eat. A typical 20 kg dog will normally experience intestinal distress after eating less than 240 g of dark chocolate, but will not necessarily experience bradycardia or tachyarrhythmia unless it eats at least a half a kilogram of milk chocolate. According to the Merck Veterinary Manual (1998), approximately 1.3 g/kg b.wt. of baker's chocolate is sufficient to cause symptoms of toxicity, e.g. a typical 25 g baker's chocolate bar would be enough to bring out symptoms in a 20 kg dog.

The negative effects depend on the dosage, the size of the dog, and the type of chocolate. LD_50_ of methylxanthines in animals are summarised in Table 2 (*www.actionagainstpoisoning.com*).

**Table 2:** LD_50_ of methylxanthines in animals (www.actionagainstpoisoning.com).

Methylxanthine	Animal	LD_50_ (mg/kg)
Theophylline	Dog	300
	Cat	700
Caffeine	Dog	140
Theobromine	Dog	250–500
	Cat	200
	Mouse	837
	Rat	1 265

### Absorption, distribution, metabolism, excretion (ADME)

Theobromine and caffeine are readily absorbed from the GI tract and are widely distributed throughout the body. They are metabolized in the liver and undergo enterohepatic recycling and then excreted in the urine as both metabolites and unchanged parent compounds. Methylxanthines also readily pass in the milk of exposed lactating animals. Very little parent compound is passed in the feces. The half-lives of theobromine and caffeine in dogs are 17.5 h and 4.5 h, respectively.

### Mechanism of action

Methylxanthines cause a competitive antagonism of cellular adenosine receptors. Inhibition of adenosine receptors causes CNS stimulation, constriction of some blood vessels, diuresis, and tachycardia. Methylxanthines also increase intracellular calcium levels by increasing cellular calcium entry and inhibiting intracellular sequestration of calcium by the sarcoplasmic reticulum of striated muscles. The net effect is increased strength and contractility of skeletal and cardiac muscle. Methylxanthines may also compete for benzodiazepine receptors within the CNS and inhibit phosphodiesterase, resulting in increased cyclic adenosine monophosphate (AMP) levels. They may also increase circulating levels of epinephrine and norepinephrine (*www.merckvetmanual.com*).

### Clinical signs

Clinical signs of toxicosis usually occur within 6–12 h of ingestion, e.g. nausea, vomiting, diarrhoea, dyspnoe, thirst, and increased urination. These can progress to dehydration, restlessness, hyperactivity, cardiac arrhythmias, internal bleeding, heart attacks, tachypnea, ataxia, tremors, seizures, weakness, coma, cyanosis, hypertension, hyperthermia, and eventually death. The high fat content of chocolate products may trigger pancreatitis in susceptible animals. In general, mild signs (vomiting, diarrhoea, polydipsia) may be seen in dogs ingesting 20 mg/kg, cardiotoxic effects may be seen at 40–50 mg/kg, and seizures may occur at doses ≥60 mg/kg (*www.merckvetmanual.com*). A dog, which ingested a lethal quantity of garden mulch made from cacao bean shells, developed severe convulsions and died 17 h later. Analysis of the stomach content and the ingested cacao bean shells revealed the presence of lethal amounts of theobromine (Drolet *et al*., 1984).

### Treatment

Once animals have stabilized, or in animals presenting before clinical signs have developed (e.g. within 1 h of ingestion), decontamination should be performed. Induction of emesis using apomorphine (0.08 mg/kg i.m. or s.c.) or hydrogen peroxide should be initiated; in animals that have been sedated due to seizures, gastric lavage may be considered. Activated charcoal (1–4 g/kg, p.o.) should be administered; because of the enterohepatic recirculation of methylxanthines, repeated doses should be administered every 3 h for up to 72 h in symptomatic animals (control vomiting with metoclopramide, 0.2–0.4 mg/kg, s.c. or i.m.). Erythromycin and corticosteroids should be avoided because they interfere with excretion of methylxanthines. For ventricular-origin tachyarrythmias lidocaine is applicated (1–2 mg/kg i.v. until effect, followed by 25–80 mg/kg/min infusion rate to effect to maintain). However, lidocaine cannot be used in cats. If lidocaine fails, metoprolol injection is preferred over propranolol, since metoprolol does not slow renal excretion of methylxanthines as propranolol can. Suggested starting dose for injectable form of either metoprolol or propranolol is 0.02–0.06 mg/kg slow i.v., which can be repeated and increased if needed. The rate of administration should not exceed 1 mg/2 min i.v. For bradycardia (less prevalent) atropine is used at 0.01–0.02 mg/kg i.m. or s.c. Artificial respiration is applicated if needed. To release seizures diazepam (0.5–2.0 mg/kg, slow i.v.) or barbiturates are applicated. Fluid diuresis may assist in stabilizing cardiovascular function and hasten urinary excretion of methylxanthines. Other treatment for symptomatic animals includes thermoregulation, correcting acid/base and electrolyte abnormalities, monitoring cardiac status via electrocardiography, and urinary catheter placement (methylxanthines and their metabolites can be reabsorbed across the bladder wall). Clinical signs may persist up to 72 h in severe cases.

## Grapes, raisins and sultanas

One of the more striking poisonings to have emerged as a potential concern over the last few years has been that of raisin poisoning in dogs. The ingested doses involved in these fatal cases ranged from 10 to 57 g of fruit per kg b. wt. There are now several reports that confirm that ingestion of these fruits can cause renal failure in dogs. Grapes contain an unknown toxin (Gwaltney-Brant *et al*., 2001; Penny *et al*., 2003; Campbell and Bates, 2003; Mazzaferro *et al*., 2004, Sutton and Campbell, 2006, Campbell, 2007).

### Mechanism of action

The toxic mechanism remains to be elucidated, and the apparent lack of a reproducible dose response relationship has led some authors to suggest this may reflect either a component of the fruits that is present in varying quantities, or the existence of an extrinsic compound that may not always be present. Individual variations in response may also occur (McKnight, 2005).

### Toxic doses

The general consensus at present is that potentially any dose should be considered a problem. Estimated amounts of grapes associated with renal injury in dogs are about 32 g/kg; amounts of raisins associated with signs range from 11–30 g/kg (*www.merckvetmanual.com*).

### Clinical signs

Clinical effects usually become apparent within 6 h of ingestion, and always within 24 h. Early signs are vomiting (in almost all cases), diarrhoea within 6–12 h of ingestion, anorexia, abdominal pain, weakness, dehydration, tremors and lethargy. Ingesta may be present in the vomitus or faeces. Polydipsia may also be apparent. Oliguric or anuric renal failure develops within 24–72 h of exposure; once anuric renal failure develops, most dogs die or are euthanized. Urinalysis may reveal proteinuria, glucosuria, microscopic haematuria and, rarely, crystalluria. Urine and blood tests will provide definitive evidence for it. Transient elevations in serum glucose, calcium, phosphorus, liver and pancreatic enzymes develop in some dogs (Eubig *et al*., 2005). It is generally agreed that prognosis in dogs with oliguria or anuria is poor.

### Necropsy

In some of the cases with fatal outcomes proximal renal tubule necrosis and renal calcification was described (Morrow *et al*., 2005).

### Therapy

Ingestion of any quantity of grapes, raisins or sultanas by a dog should be considered treatable. Digestion of the fruits appears to be slow and decontamination several hours post-ingestion may be worthwhile as whole grapes and swollen raisins have been recovered after remaining in the stomach overnight. Gut decontamination should be considered by means of emesis or gastric lavage. Emesis can be induced with 3% hydrogen peroxide (2 ml/kg; no more than 45 ml), followed by activated charcoal. Activated charcoal may be of benefit, but care should be taken to ensure bowel sounds are regular before this is administered. If spontaneous vomiting is protracted it must be consider to use anti-emetics such as metoclopramide (0.5–1 mg p.o., s.c. or i.m. 6–8 h or 1–2 mg/kg per day by slow i.v. injection). Aggressive i.v. fluid therapy for at least 48 h for rehydration and support of renal function is important (Campbell, 2007). Renal function and electrolytes should be monitored for at least 72 h post-ingestion. Where necessary the use of furosemide (2 mg/kg i.v. initially followed by i.v. infusion of 5 mg/kg/h), dopamine (0.5–3 μg/kg/min, i.v.) or mannitol (0.25–0.5 g/kg i.v. over 5–10 min) may be considered to re-establish urine output in oliguric dogs (McKnight, 2005). However, the efficacy of these therapies remains unproven and that there is evidence that tubular necrosis or renal tubule obstruction may prevent urine flow. Anuric dogs are unlikely to survive unless peritoneal dialysis or hemodialysis is performed, and even then the prognosis is guarded.

## Onion toxicosis

The onion (*Allium cepa*) is one of the oldest crops. Its world production has increased by at least 25% over the past 10 years with current production being around 44 million tonnes making it the second most important horticultural crop after tomatoes. Because of its storage characteristics and durability for shipping, onion has always been traded more widely than most vegetables. Onion is versatile and is often used as an ingredient in many dishes and is accepted by almost all traditions and cultures. Onion consumption is increasing significantly and this is partly because of heavy promotion that links flavour and health. Onion is rich in two chemical groups that have perceived benefits to human health – flavonoids and alk(en)yl cysteine sulphoxides. Apart from its culinary uses – fresh, cooked or dehydrated – medicinal properties have been attributed to both since ancient times, prompting in recent years an accurate chemical analysis of the most characteristic active ingredients. Compounds from onion have a range of health benefits which include anticarcinogenic properties, antiplatelet, antithrombotic activity, antiasthmatic, antidiabetic, hypocholesterolaemic, fibrinolytic and various other biological actions, and antibiotic effects (Augusti, 1996; Briggs *et al*., 2001; Griffiths *et al*., 2002; Slimestad *et al*., 2007). On the other hand, onion contains the toxic components which can damage red blood cells and cause haemolytic anaemia accompanied by the formation of Heinz bodies in erythrocytes of animals (Desnoyers, 2000), e.g. in cattle (Carbery, 1999; Rae, 1999; Van Der Kolk, 2000), horses (Thorp and Harshfielf, 1939), dogs (Stallbaumer, 1981; Harvey and Rackear, 1985; Solter and Scott, 1987; Yamato *et al*., 2005) and cats (Kobayashi, 1981).

Heinz body anaemia is an uncommon finding in dogs, and few other toxicants, e.g. methylene blue, acetaminophene, zinc, benzocaine, vitamin K, phenylhydrazine (Houston and Myers, 1993) can induce it, so onion ingestion must always be suspected in such situations.

### Mechanism of action

Various factors are implicated in the wide variation in species susceptibility, including differences in hemoglobin structure and protective enzyme systems. The mechanism of onion toxicity has been known for several decades, but recent studies have shown that more than one toxin is involved. The toxic components in all type of onions, garlic, leeks, shallots, and other plants of the *Allium* family, are sulfoxides and aliphatic sulfides, specifically allyl and propyl di-, tri-, and tetrasulfides. Onions contain also the relatively rare amino acids S-meth and S-prop (en)ylcysteine sufoxides (SMCO). It is widely agreed that n-propyl disulphide is the principal toxin that reduces the activity of glucose-6-phosphate dehydrogenase in red blood cells; thereby interfering with regeneration of reduced glutathione needed to prevent oxidative denaturation of haemoglobin (Thrall, 2004). Denaturated haemoglobin, when developed, precipitates on the surface of red blood cells (named Heinz bodies) and triggers intra- and extravascular haemolysis (Lincoln *et al*., 1992; Tang *et al*., 2008). The haemolytic effect of sodium n-propylthiosulphate, which had been isolated from boiled onions, was studied in dogs by Yamato *et al*. (1998). The oral administration of 500 mumol/kg b. wt. of the compound to dogs resulted in a haemolytic anaemia associated with an increase of Heinz body formation in erythrocytes, which was more severe in dogs with the hereditary condition which results in erythrocytes with high concentrations of reduced glutathione and potassium than in normal dogs. In the affected dogs there was a 10-fold increase in the concentration of oxidised glutathione in their erythrocytes 12 h after the administration of the compound, whereas in normal dogs there was almost no change.

### Toxicity

Consumption of as little as 5 g/kg of onions in cats or 15 to 30 g/kg in dogs has resulted in clinically important hematologic changes (Cope, 2005). Onion toxicosis is consistently noted in animals that ingest more than 0.5% of their b. wt. in onions at one time. A relatively high dosage (600–800 g) in one meal or spread apart over a few days can damage red blood cells and cause haemolytic anaemia accompanied by the formation of Heinz bodies in erythrocytes.

All forms of onion can be a problem including: dehydrated, raw or cooked onions, table scraps containing cooked onions or garlic, left over pizzas, chinese dishes, any feeding stuff containing onions.

Humans are the most resistant species studied. On the other hand, there is some concern about the susceptibility of certain ethnic groups that have a genetic deficiency of glucose-6-phosphate dehydrogenase.

Although the dog appears to be one of the most susceptible species, there are very few reports in the scientific literature concerning accidental canine poisoning associated with onion ingestion. Cats are more susceptible than dogs. Since baby food is often used in sick cats that are not eating (to stimulate their appetite), there was concern that the onion powder would cause a Heinz body anemia in these cats (Robertson *et al*., 1998). Many baby food manufacturers add onions or onion powder to increase palatability. It is generally accepted that sheep, goats, rats and mice are more resistant to onion toxicosis than other domestic animals (Thrall, 2004; Aslani *et al*., 2005). The safety of feeding culled onions to livestock depends upon species susceptibility and the toxic potential of the onions. Sheep can be maintained on diets of up to 50% onions with no clinical abnormalities or detrimental effects on growth. Even when onions are fed free choice, sheep have only transient hemoglobinuria and anemia, with few deaths even reported. In contrast, cattle should be fed onions with caution, due to the relative susceptibilty of their erythrocytes to oxidative damage. Daily feeding of onions could have a cumulative effect due to ongoing formation of Heinz bodies versus a single exposure with a wide gap until the next exposure, allowing the bone marrow time to regenerate the prematurely destroyed red cells.

### Clinical signs

The first symptoms are usually of gastro-enteritis: vomiting, diarrhoea, abdominal pain, loss of appetite, depression and dehydration. It will take a few days for the dog to display the symptoms associated with the loss of red blood cells: pale mucous membranes, rapid respiratory rate, difficulty to breath, lethargy, dark coloured urine – reddish or brown, jaundice, weakness, rapid heart rate. Haematology may reveal neutrophilia, lymphopenia, Heinz-body anaemia and methaemoglobinaemia.

### Therapy

There is not any antidote, however, supportive care may be helpful: hospitalisation, administration of intra-venous fluids, blood transfusions. Treatment is advocated of ingestion of any quantity. For recent ingestions gastric decontamination should be considered, and use of adsorbents, but thereafter management is largely supportive. It is important that the animals remain hydrated; antiemetics may be given to control persistent vomiting. Nonenzymatic reductants such as ascorbic acid may also be useful (in dog 30 mg/kg b. wt. i.v. each 6–8 h). Antioxidant prevention (by N-acetylcysteine, vitamin E, ascorbate) of Heinz body formation and oxidative injury in cats was recommended by Hill *et al*. (2001). In severely poisoned animals blood transfusions have been successfully employed.

Nevertheless, even taking into account that lethal effects are infrequent in dogs, avoiding exposure to any kind of *A. cepa* in this and other domestic animal species seems to be the best preventive health strategy.

## Garlic

Garlic (*Allium sativum*) is considered to be less toxic and safe for dogs than onion when used in moderation. Allicin and ajoene, pharmacologically active agents in garlic, are potent cardiac and smooth muscle relaxants, vasodilators, and hypotensive agents (Malik and Siddiqui, 1981; Mayeux *et al*., 1988; Martin *et al*., 1992). Lee *et al*. (2000) studied wether dogs given garlic extract developed hemolytic anemia. Garlic extract was administered intragastrically (1.25 ml/kg of b.wt. (5 g of whole garlic/kg) once a day for 7 days). Compared with initial values, erythrocyte count, haematocrit and hemoglobin concentration decreased to a minimum value on days 9 to 11. Heinz body formation, an increase in erythrocyte-reduced glutathione concentration, and eccentrocytes were also detected, however, no dog developed hemolytic anemia. Eccentrocytosis appears to be a major diagnostic feature of garlic-induced hemolysis in dogs (Lee *et al*., 2000; Yamato *et al*., 2005).

Garlic is toxic also for horses, at a daily dose of >0.2 g/kg causes Heinz body anemia in them (Pearson *et al*., 2005).

## Avocado

Avocado fruit, pits, leaves and the actual plant are all potentially poisonous to dogs, along with other pets like cats, mice, rats, birds, rabbits, horses, cattle and goats, among others.

Persin is a fungicidal toxin found in both the fruit and leaves of the avocado tree (*Persea americana*). It has been isolated only recently and discovered to kill breast cancer cells. It has also been shown to enhance the effect of the breast cancer fighting drug Tamoxifen. This could potentially reduce the necessary dosage of current cancer drugs. Persin is however highly insoluble, and more research will be needed to put it into a soluble tablet form.

Feeding avocados to any non-human animal should be completely avoided. The lethal dose is not known; the effect is different depending upon the animal species. Avocados will trigger fluid accumulation in the lungs and chest, leading to difficulty breathing and death due to oxygen deprivation. Fluid accumulation can also occur in the heart, pancreas and abdomen (Buoro *et al*., 1994). High fat content of avocado can lead to pancreatitis.

### Clinical signs

The symptoms include gastrointestinal irritation, vomiting, diarrhoea, respiratory distress, congestion, fluid accumulation around the tissues of the heart and even death. Birds seem to be particularly sensitive to this toxic compound and the symptoms are the increased heart rate, myocardial tissue damage, labored breathing, disordered plumage, unrest, weakness, and apathy. High doses cause acute respiratory syndrome (asphyxia), with death approximately 12 to 24 h after consumption. In lactating rabbits and mice non-infectious mastitis and agalactia after consumption of leaves or bark was observed. In rabbits cardial arrhythmia, submandibular edema and death after consumption of leaves occur. In cows, horses and goats mastitis after consumption of leaves or bark was observed.

## Macadamia nuts

Macadamia nuts originate from the trees *Macadamia integrifolia* in the continental USA and *Macadamia tetraphylla* in Hawaii and Australia. They are commonly present in cookies, so owners should be careful what they feed their dog. Macadamia nuts (both raw and roasted) as well as macadamia butter contain an unknown toxin that affects the muscles, digestive system and nervous system and can cause locomotory difficulties, weakness, dyspnoe, tremors and swollen limbs. Macadamia nuts and walnuts can also trigger pancreatitis and peanuts a deadly allergic reaction (Knott *et al*., 2008).

### The mechanism of action

The mechanism of toxicity is unknown; but may involve a constituent of the nuts, processing contaminants or mycotoxins.

### Toxicity

The toxic dose to dogs ranges from 2.4–62.4 g per kg b.wt. This is a very large range and can mean that some dogs will get ill with just a small amount of nuts ingested, while other dogs need to eat a lot of nuts to show signs (Hansen *et al*., 2000).

### Clinical signs

Dogs are the only species in which signs have been reported (*www.merckvetmanual.com*). Within 12 h of ingestion, dogs develop weakness (more pronounced in hind limbs), depression, vomiting, abdominal pain, ataxia, muscle tremors, swollen and painful limbs, paralysis of the hindquarters, hyperthermia (with rectal temperatures up to 40.5 °C), an elevated heart rate, lameness, stiffness and recumbency. Tremors may be secondary to muscle weakness. Macadamia nuts may be identified in vomitus or feces. Mild transient elevations in serum triglycerides, lipases, and alkaline phosphatase were reported in some dogs experimentally dosed with macadamia nuts; these values quickly returned to baseline by 48 h after experimental dosing (Hansen *et al*., 2000). Signs generally resolve within 12–48 h (Hansen *et al*., 2000; Hansen, 2002). The onset of clinical signs was reported as <12 h in 79% of the cases. The duration of clinical signs for the majority of cases was <24 h. The amount of macadamia nuts ingested was estimated in 72% of the calls with a mean of 11.7 g/kg bw. All field and experimental dogs recovered uneventfully within 1 to 2 d whether treated by a veterinarian or not. No mortality has been reported.

### Therapy

For asymptomatic dogs with recent ingestion of more than 1–2 g/kg, emesis should be induced; activated charcoal may be of benefit with large ingestions. Fortunately, most symptomatic dogs will recover without any specific treatment. Severely affected animals may be given supportive treatment such as fluids, analgesics or antipyretics (*www.merckvetmanual.com*). Use of mild laxatives may assist the passage of ingesta through the gastrointestinal tract. Luckily, the muscle weakness, while painful, seems to be of short duration and the patients do recover from the toxicity.

## Effects of xylitol ingestion

Xylitol, an artificial sweetener, is present in many products, such as candy, sugar-free chewing gums, toothpaste and baked goods. Ingestion of these foods by dogs results in a significant, and often sustained, insulin-mediated hypoglycemic crisis (Cope, 2004). In both humans and dogs, the levels of blood sugar are controlled by the body's release of insulin from the pancreas. In human xylitol ingestion does not cause any significant changes in insulin levels or, therefore, blood glucose (Dunayer, 2004). However, in dogs, xylitol is a strong promoter of insulin release, which results in a rapid decrease in blood glucose (hypoglycemia) (Asano *et al*., 1977; Dunayer, 2004; Campbell and Bates, 2008). A sudden drop in blood sugar then results in depression, ataxia, seizures and collapse (Thomas and Boag, 2008). This compound can cause liver damage and death. This information was first published in July 2004.

### Clinical signs

Clinical signs of xylitol toxicity can develop in as few as 30 min after ingestion and may include one or more of the following: vomiting, weakness, ataxia, depression, hypokalemia, seizures, coma, liver dysfunction and/or failure. Dunayer and Gwaltney-Brant (2006) observed in dogs lethargy and vomiting after ingestion of xylitol. In addition some dogs had widespread petechial, ecchymotic, or gastrointestinal tract hemorrhages, moderately to severely high serum activities of liver enzymes, hyperbilirubinemia, hypoglycemia, hyperphosphatemia, prolonged clotting times, and thrombocytopenia. Necropsy revealed severe hepatic necrosis, hepatocyte loss or atrophy with lobular collapse.

### Treatment

The induction of vomiting is recommended if performed very soon after ingestion of the xylitol-containing product but before clinical signs develop. Frequent small meals or an oral sugar supplement may be used to manage dogs that have not yet shown clinical signs. Following the appearance of clinical signs intravenous dextrose can be used to control hypoglycemia. It may also be necessary to treat the patient for hypokalemia, if indicated. Treatment should be continued until the blood glucose levels return to normal levels.

Dunayer and Gwaltney-Brant (2006) recommended for treatment i.v. administration of fluids; plasma transfusions; and, if indicated, administration of dextrose. Cope (2004) revealed that binding of xylitol to activated charcoal is relatively low; however, activated charcoal administration may still be beneficial in some canine acute oral xylitol exposures.

## Alcohol toxicoses

Serious intoxications have occurred when dogs have been given alcohol to drink as a „joke“. Also, dogs seem to be attracted to alcoholic drinks, so drinks should not be left unattended. Dogs cannot tolerate alcohol, even in small amounts.

Ethanol is the alcohol in alcoholic beverages, perfumes and mouthwashes. Ethanol toxicosis in dogs occurred after ingestion of rotten apples (Kammerer *et al*., 2001), alcoholic beverages (van Wuijckhuise and Cremers, 2003) or uncooked bread dough that contains *Saccharomyces cerevisiae* (brewer's and baker's yeast), which metabolizes carbohydrate substates to ethanol and carbon dioxide (Thrall *et al*., 1984; Suter, 1992; Means, 2003). Ethanol toxicosis can also occur in dogs and cats after overdosage when ethanol is given intravenously as a compatitive substrate to treat ethylene glycol toxicosis (Thrall *et al*., 1998).

### Mechanism of action

The mechanism of action on the CNS is related in part to its interactions with biomembranes and its probable inhibition of gamma-amino butyric acid (GABA) receptors (Valentine, 1990).

### Clinical signs

Clinical signs include ataxia, lethargy, sedation, hypothermia, metabolic acidosis, vomiting, diarrhoea, poor breathing, liver failure, coma, and death. And the hops in beer are also potentially toxic to dogs and cause dyspnoe, increased heart rate, elevated temperature, seizures and death.

### Therapy

Severe ethanol intoxication requires mechanical ventilation. Electrolyte and acid-base status should be also corrected (Richardson, 2006).

## Conclusion

There are also other human foods that may cause intoxications in pets, e.g. tomato, pottato, rhubarb, persimons, etc. **The best advice must surely be to give animals foodstuffs and or treats specifically developed for their diets.**
